# Comparison and analysis of multiple machine learning models for discriminating benign and malignant testicular lesions based on magnetic resonance imaging radiomics

**DOI:** 10.3389/fmed.2023.1279622

**Published:** 2023-12-21

**Authors:** Yanhui Feng, Zhaoyan Feng, Liang Wang, Wenzhi Lv, Zhiyong Liu, Xiangde Min, Jin Li, Jiaxuan Zhang

**Affiliations:** ^1^School of Medicine and Health Management, Huazhong University of Science and Technology, Wuhan, China; ^2^Department of Radiology, Tongji Hospital, Tongji Medical College, Huazhong University of Science and Technology, Wuhan, China; ^3^Computer Center, Tongji Hospital, Tongji Medical College, Huazhong University of Science and Technology, Wuhan, China; ^4^Britton Chance Center and MoE Key Laboratory for Biomedical Photonics, Wuhan National Laboratory for Optoelectronics, Huazhong University of Science and Technology, Wuhan, China

**Keywords:** boosting algorithm, machine learning, magnetic resonance imaging, radiomics, testicular neoplasms

## Abstract

**Objective:**

Accurate identification of testicular tumors through better lesion characterization can optimize the radical surgical procedures. Here, we compared the performance of different machine learning approaches for discriminating benign testicular lesions from malignant ones, using a radiomics score derived from magnetic resonance imaging (MRI).

**Methods:**

One hundred fifteen lesions from 108 patients who underwent MRI between February 2014 and July 2022 were enrolled in this study. Based on regions-of-interest, radiomics features extraction can be realized through PyRadiomics. For measuring feature reproducibility, we considered both intraclass and interclass correlation coefficients. We calculated the correlation between each feature and the predicted target, removing redundant features. In our radiomics-based analysis, we trained classifiers on 70% of the lesions and compared different models, including linear discrimination, gradient boosting, and decision trees. We applied each classification algorithm to the training set using different random seeds, repeating this process 10 times and recording performance. The highest-performing model was then tested on the remaining 30% of the lesions. We used widely accepted metrics, such as the area under the curve (AUC), to evaluate model performance.

**Results:**

We acquired 1,781 radiomic features from the T2-weighted maps of each lesion. Subsequently, we constructed classification models using the top 10 most significant features. The 10 machine-learning algorithms we utilized were capable of diagnosing testicular lesions. Of these, the XGBoost classification emerged as the most superior, achieving the highest AUC value of 0.905 (95% confidence interval: 0.886–0.925) on the testing set and outstripping the other models that typically scored AUC values between 0.697–0.898.

**Conclusion:**

Preoperative MRI radiomics offers potential for distinguishing between benign and malignant testicular lesions. An ensemble model like the boosting algorithm embodied by XGBoost may outperform other models.

## 1 Introduction

Testicular cancer, which is the most common solid tumor in males aged 15–34 years, is anticipated to result in approximately 470 fatalities and usher in an estimated 9,190 new cases in the United States by 2023 (1). Based on a 2020 statistical report, testicular cancer ranks among the top five causes of cancer-related fatalities in males aged 20–39 years in the United States (2). The standard treatment for malignant testicular lesions is inguinal orchiectomy (3, 4). For patients with benign testicular lesions, a more sensible treatment approach often involves conservative care, complemented by regular follow-ups and testicular preservation surgery. This is primarily because orchiectomy can adversely impact the patient’s reproductive abilities and mental health, an effect particularly profound among young adult males (4, 5). Accurate identification of testicular tumors through better lesion characterization can help to reduce unnecessary radical surgical procedures (6).

Ultrasonography (US) is often used to confirm the presence of tumors in patients with testicular lesions (7). However, US has limited ability to distinguish benign and malignant testicular lesions effectively or to predict tumor size accurately (8). The advanced multi-directional and multi-sequence scanning capabilities of magnetic resonance imaging (MRI) can effectively depict testicular lesions and their relationship to surrounding tissues. Furthermore, it can infer possible tissue compositions, thereby providing valuable aid in both the diagnosis and differential diagnoses of these lesions (9). Therefore, MRI can afford us more adequate information and help to clarify some uncertainties or ambiguities in the results of the US, thereby reducing unnecessary surgical treatment (10).

Machine learning (ML), a multidisciplinary facet of artificial intelligence, endows computers with the capability to learn, enabling them to perform complex tasks similarly to humans. It is applied to both scientific research and industrial production to make accurate predictions using diverse data sources (11). Since it has achieved excellent prediction results in a wide range of applications, machine learning technology has attracted significant interest from medical researchers and clinicians (12).

In the past decade, the rapid development of medical image analysis has promoted the development of radiomics, which acquires massive quantitative information from image (13–15). It has a great application prospect in diagnosis, grading, staging, and prognosis of many tumors (16–18). Our previous studies established machine learning using radiomic signatures based on histogram analysis of apparent diffusion coefficient (ADC) (19). A previous study combined features and clinical indicators extracted from MRI to create predictive models to diagnose benign and malignant testicular lesions (20). However, to the best of our knowledge, no study to date has compared different modeling methods for the diagnosis of testicular diseases.

Therefore, we intend to utilize MRI imaging data for a comparative analysis of various machine learning algorithms deployed in differentiating between benign and malignant testicular diseases.

## 2 Materials and methods

### 2.1 Patients

**Figure 1:**
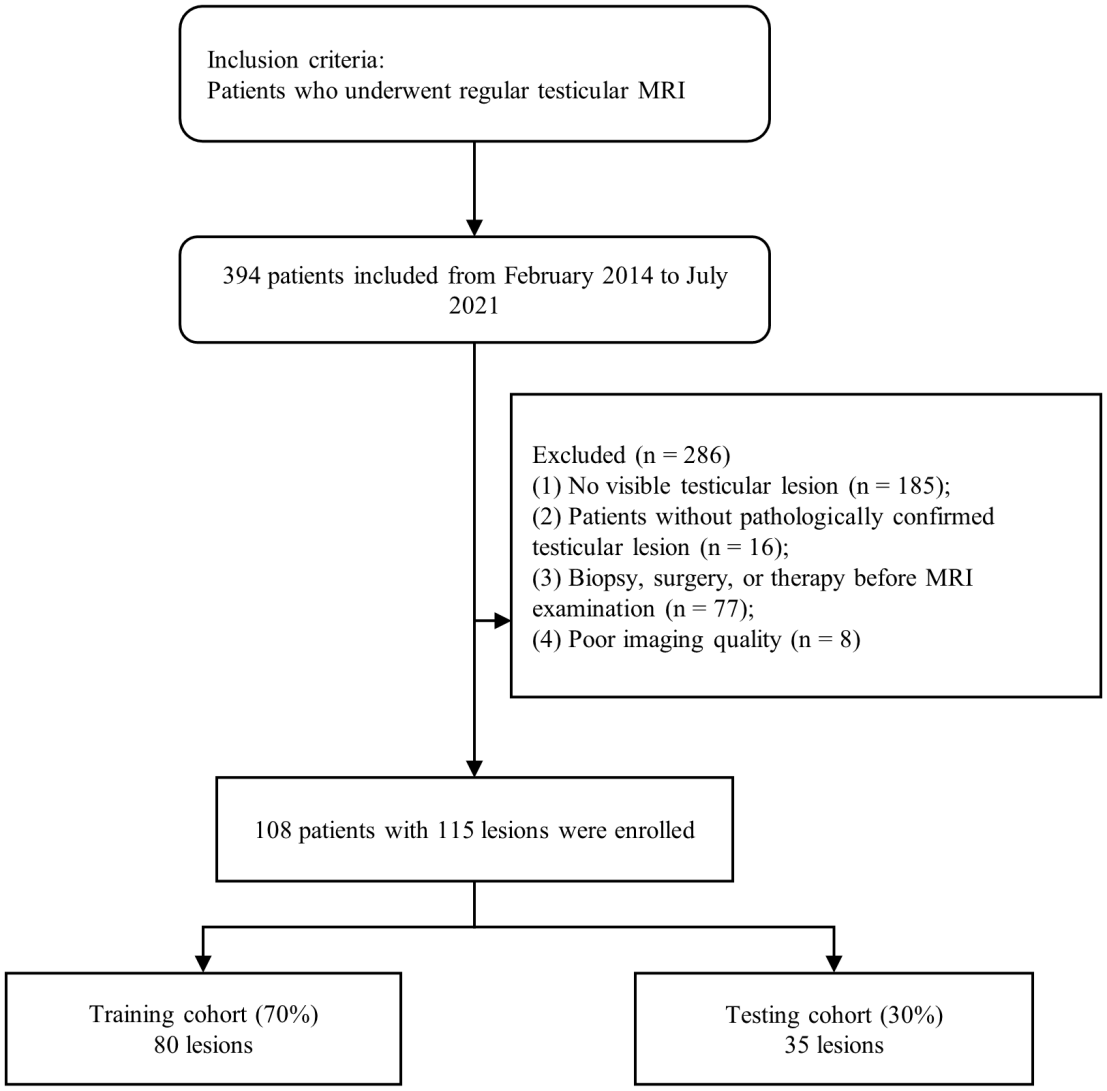
Inclusion and exclusion criteria.

A total of 394 patients, who underwent routine testicular MRI examinations, were recruited from February 2014 to July 2022. Of these, 286 patients were excluded based on the following criteria: (1) patients with no significant testicular lesions on MRI (*n* = 185); (2) patients who underwent biopsies, surgery, or treatment prior to MRI examination (*n* = 77); (3) patients with no testicular lesions confirmed by pathology (*n* = 16); and (4) patients who lacked MRI data or had MRI data of poor image quality (*n* = 8). Finally, 115 lesions were identified from 108 patients screened, including 44 benign and 71 malignant tumors. In this study, all lesions were diagnosed from testicular tissue sections after surgery or biopsy specimens. A flowchart of the case identification process is shown in Figure 1.

### 2.2 MRI protocol

We use the advanced type superconducting magnetic resonance system MAGNETOM Skyra, to scan patients with follow specification 3.0 T technology parameters, and set up an 18-element matrix and a 32-channel coil. The MRI protocol was listed in Supplementary Table S1. Due to the limited sample size, diffusion-weighted imaging and dynamic contrast-enhanced MRI were not included in this study.

### 2.3 Image segmentation

All transverse T2-weighted images (T2WI) were input into ITK-SNAP software (version 3.4.0) to realize the 3D segmentation of the target region manually. The lesions of all patients were manually segmented by radiologists with extensive experience in abdominal imaging. The two readers had 4 years and 5 years of experience, respectively. Segmentation was independently conducted to assess the reproducibility of inter-observer segmentation. Both two readers were blinded to the histopathological results. A radiologist with 4 years of experience (Reader 1) visualized the testicular lesions 1 month later to assess intra-observer segmentation reproducibility.

### 2.4 Radiomics feature extraction

The PyRadiomics package (version 2.1.2) was adopted to extract features from MRI. All MRI data were resampled with the same resolution (1.0 × 1.0 × 1.0 mm), and the built-in standardization function of PyRadiomics with a scale of 1 was used to normalize the intensity of MRI data. Nineteen filters were applied to each MRI scan of a lesion, as listed in Supplementary Table S2. All classes of features (Supplementary Table S3), with the exception of shape, were computed for both the original and derived images.

### 2.5 Inter- and intra-correlation analysis of features

**Figure 2:**
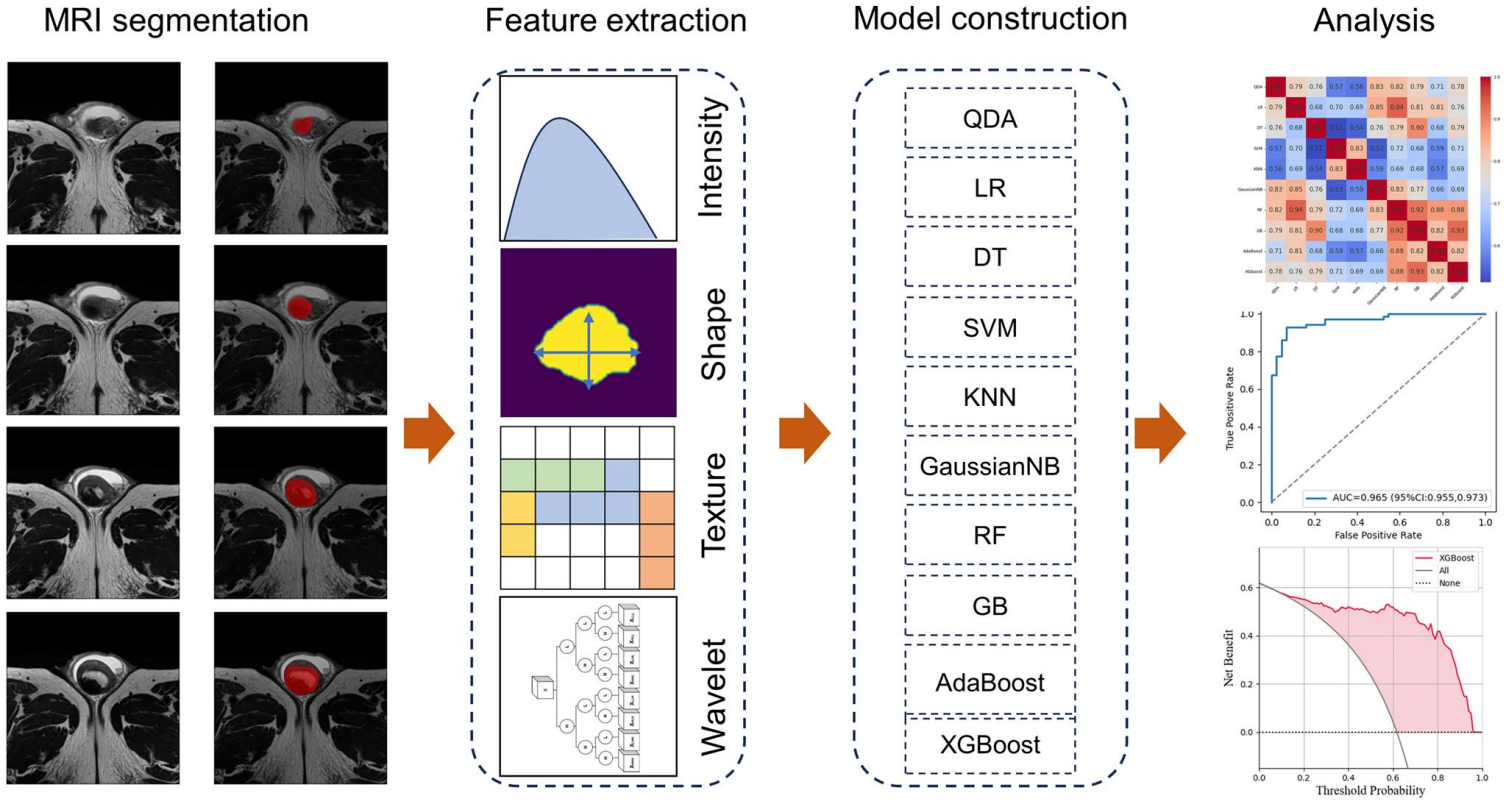
Illustration of the study design.

The robustness of the features was evaluated using ICCs. Randomly selected 34 lesions and the segmentation was operated by Reader 1 (4 years’ experience in abdominal imaging). Secondary segmentation of these cases was performed by Reader 1 month later to evaluate the reproducibility within the observer. These images were also assessed by Reader 2 (5 years’ experience in abdominal imaging) to assess consistency between observers. Features with ICC ≥0.8 were considered to be robust and were included in the follow-up study. Feature selection was performed with the maximum relevance and minimum redundancy (mRMR) approach (21), and the classification model based on radiomics was established. Figure 2 shows the workflow of radiomics signature development.

### 2.6 Model construction and evaluation

The included cases were divided into the training and testing set according to the ratio of 7:3. The following machine-learning models were considered: logistic regression (LR), quadratic discriminant analysis (QDA), *k*-nearest neighbor classifier (KNN), decision tree (DT), support vector machine (SVM), Gaussian naive Bayes (GaussianNB), random forest (RF), adaptive boosting (AdaBoost), gradient boosting (GB), and extreme gradient boosting (XGBoost). In the training set used to evaluate prediction performance and stability, different random seeds were set to train each classifier for 10 times. The average performance on the training set was recorded (Supplementary Table S4). The optimal model in the training cohort was subsequently tested in testing set.

When using the XGBoost algorithm, the following parameters are considered for adjustment: The learning_rate refers to the learning rate or step size, which controls the adjustment of model weights in each iteration. A small learning rate may require more training rounds, but it can potentially result in better prediction performance. The n_estimators refers to the number of trees, i.e., the number of sub-models or subtrees in the generated model. Insufficient trees may cause underfitting, while an excess of trees may cause overfitting. The max_depth indicates the maximum distance between the root node and the furthest leaf node in each tree. It affects the complexity of the model, as deeper trees result in a more complex model. Excessively large depths can lead to overfitting. The min_child_weight is used to determine the minimum weight sum of child sub-trees. If the weight of instances in a newly partitioned sub-tree is below this value, further partitioning will not occur. This parameter helps avoid overfitting. The gamma parameter adjusts the degree of instance importance. A node will only split if the reduction in the loss function value after the split exceeds the specified gamma threshold. The colsample_bytree refers to the subsample ratio of columns, which is feature sampling used to construct each tree. The colsample_bytree is the subsample ratio considered during tree building. The subsample represents the subsample ratio of observed samples, which helps prevent overfitting. Typically, the value is between 0.5 and 1. In the experimental phase of this study, grid search was used to find appropriate parameters that ensured the model maintained optimal performance.

### 2.7 Statistical analysis

The Python (version 3.7) package was used for statistical analysis. For continuous variables, data are presented as means ± standard deviation. ICCs were computed to evaluate the agreement between features. Indicators covered the area under the receiver operating characteristic curve (AUC), average precision of the curve and five confusion matrix related indicators. These were computed by the bootstrap method (1,000 subsamples, 100 times). To evaluate the efficient of models and clinical practicability, calibration curve and decision curve analysis (DCA) analyses were employed. *p*-values less than 0.05 were considered to be statistically significant.

## 3 Results

### 3.1 Patients

After inclusion and exclusion and characteristic analysis, the study included108 patients with 115 testicular lesions (44 benign and 71 malignant). Patients had a wide age range (from 5 to 74 years), and the mean age was 36.25 years. Besides, the mean ages of the patients with benign and malignant lesion were 33.93 years and 46.40 years, respectively. Pathological analysis was performed in each case, and the statistical distribution is presented in Table 1. No significant difference in age was observed between the benign and malignant groups (*p* = 0.217).

**Table 1:** Distribution of pathological findings in the included cases.

Benign		Malignant	
**Mean age (years)**	33.90 ± 17.00	**Mean age (years)**	46.40 ± 11.73

Pathology	Patient number	Lesion number	Pathology	Patient number	Lesion number
Epidermoid cyst	12	12	Seminoma	25	25
Sex cord-mesenchymal tumor	8	8	Embryonal carcinoma	9	9
Infection	13	15	Mixed germinoma	10	10
Cyst	1	1	Lymphoma	11	14
Infarction	1	1	Teratoma	6	6
Contusion	3	3	Testicular metastases	3	4
Prepubertal teratoma	1	1	Neuroendocrine carcinoma	1	1
Prepubertal teratoma and epidermoid cyst	1	1	Yolk sac tumor	1	1
Testicular adrenal rest tumor	1	2	Borderline serous tumor	1	1
Summation	41	44		67	71

### 3.2 Radiomics feature extraction and selection

T2WI contrast-enhanced sequence was used for radiomics features extraction. For each image space, 356 non-texture and 1,425 texture features were obtained from both the original and filtered images. ICCs were calculated for the inter-observer agreement, and 1,277 and 1,242 features were thought to be highly reproducible in terms of ICC values (ICC ≥0.8). A total of 1,182 features were considered to be robust and were included in the subsequent analysis. Finally, the mRMR method was used to eliminate redundant features and to select a subset of 10 features that were most relevant to the target to build the classification models. The radiomics features ranked by the mRMR method were mostly filter-based (7/10), which played an important role in the establishment of models.

### 3.3 Performance of models

On the training set, the prediction performance of 10 machine learning models was evaluated. All models performed well on the training set (AUC scores were greater than 0.8), and their performances are listed in Table 2. Among all the models, XGBoost exhibited the best diagnostic performance, which has a highest AUC (0.905, 95% CI, 0.886–0.925), sensitivity (0.895, 95% CI, 0.867–0.928), accuracy (0.886, 95% CI, 0.864–0.908), and NPV (0.875, 95% CI, 0.844–0.901) on the testing set. Other indicators of performance are showed in Table 3.

**Table 2:** Performance of the models in the training cohort.

Model	AUC	Sensitivity	Specificity	Accuracy	Average precision	NPV	PPV
QDA	0.905	0.654	**1.000**	0.775	0.952	0.609	**1.000**
LR	0.826	0.654	0.893	0.738	0.908	0.581	0.919
DT	0.796	0.923	0.643	0.825	0.822	0.818	0.828
SVM	0.897	0.904	0.786	0.863	0.937	0.815	0.887
KNN	0.852	0.538	**1.000**	0.700	0.894	0.538	**1.000**
Gaussian NB	0.828	0.731	0.821	0.763	0.903	0.622	0.884
RF	0.911	0.865	0.821	0.850	0.954	0.767	0.900
GB	0.936	0.788	0.964	0.850	0.964	0.711	0.976
AdaBoost	0.923	0.712	**1.000**	0.813	0.954	0.651	**1.000**
XGBoost	**0.987**	**0.942**	0.964	**0.950**	**0.994**	**0.900**	0.980

The bold values indicate the highest values for specific indicators across different models. AUC, area under the curve; CI, confidence interval; NPV, negative predictive value; PPV, positive predictive value.

**Table 3:** Performance of XGBoost in the testing cohort.

Group	AUC (95% CI)	Sensitivity (95% CI)	Specificity (95% CI)	Accuracy (95% CI)	Average precision (95% CI)	NPV (95% CI)	PPV (95% CI)
Training set	0.987 (0.982, 0.991)	0.942 (0.928, 0.958)	0.964 (0.941, 0.981)	0.950 (0.938, 0.962)	0.994 (0.992, 0.996)	0.900 (0.877, 0.926)	0.980 (0.968, 0.990)
Testing set	0.905 (0.886, 0.925)	0.895 (0.867, 0.928)	0.875 (0.848, 0.904)	0.886 (0.864, 0.908)	0.934 (0.922, 0.946)	0.875 (0.844, 0.911)	0.895 (0.874, 0.917)

AUC, area under the curve; CI, confidence interval; NPV, negative predictive value; PPV, positive predictive value.

**Figure 3:**
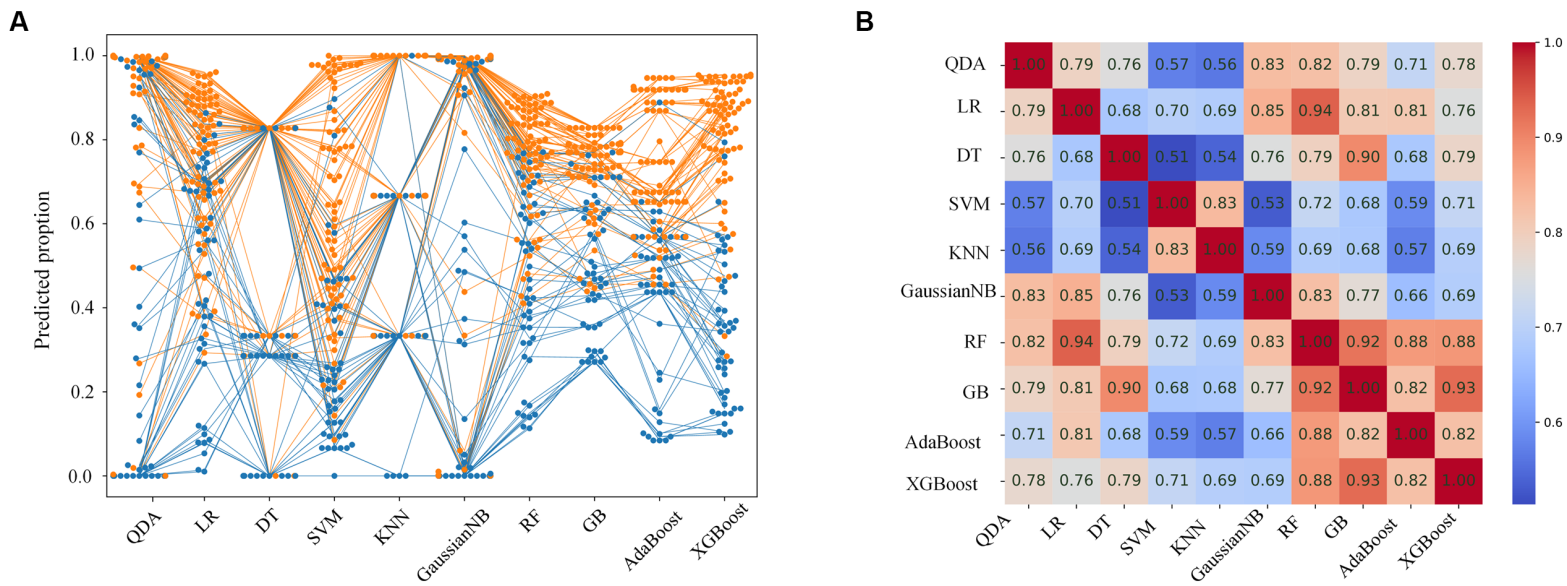
Prediction probabilities and correlation coefficients for each model. **(A)** Swarm plot of predicted probability of each model for all cases. Each dot represents a single sample. The orange and blue dots indicate malignant and benign lesions, respectively. **(B)** Correlation coefficients of the predicted probability for each model.

The prediction probabilities of each model for all lesions are shown in Figure 3A. The positive cases are mainly concentrated at the top, whereas the negative samples are mainly at the bottom, and the predicted results are more consistent with the reality. However, cases with a prediction probability of about 0.5 are relatively difficult to estimate, and the predicted values of each model are scattered. The correlation coefficients of the probabilities for each model are showed in Figure 3B. The coefficients of the RF, GB, AdaBoost and XGBoost models were 0.82 or higher (range: 0.82–0.93), indicating strong correlations. In addition, the LR, DT, GaussianNB, and RF models had high correlations, with coefficients >0.82, particularly RF and LR (coefficient = 0.94), while the correlation coefficient of SVM and KNN was 0.83.

**Figure 4:**
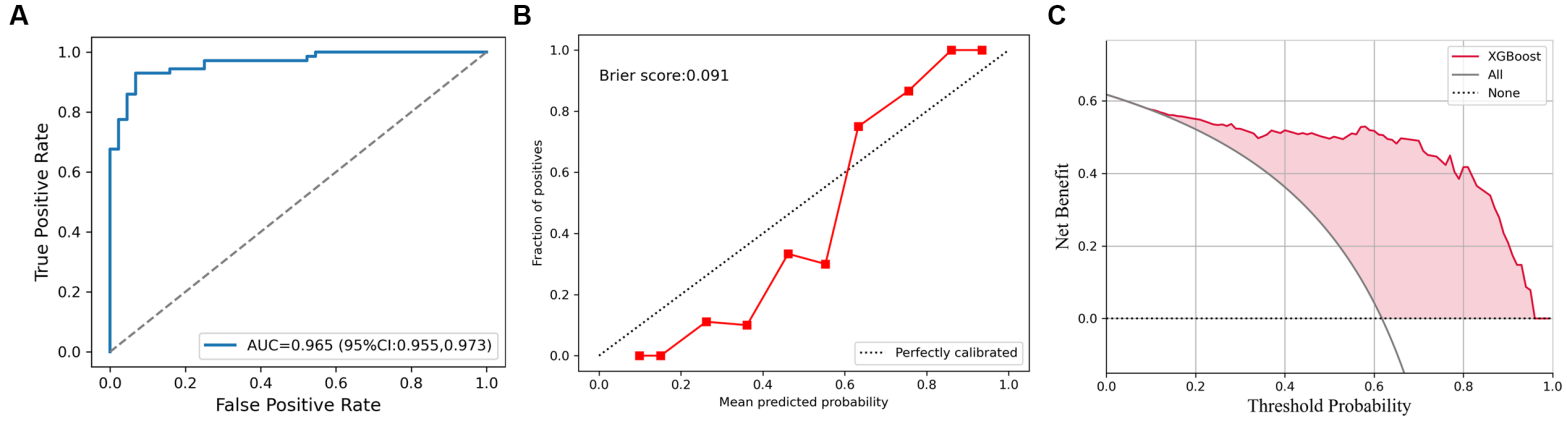
The XGBoost classifier for all cases. **(A)** Receiver operating characteristic (ROC) curve for XGBoost for discrimination of testicular lesions. **(B)** Calibration curve shows that the possibility of malignant testicular tumors is consistent with the true incidence. **(C)** Decision curve analysis (DCA) plot of the testicular lesions.

In all cases, the AUC of XGBoost was 0.965 (95% CI, 0.955–0.973), as shown in Figure 4A. The Brier score of calibration curve is 0.091 (Figure 4B), which means the predicted probability and the actual malignant testicular lesions are approximated. In the decision curve, compared to assuming that all testicular tumors are malignant, the net profit of the prediction using XGBoost will be higher between the prediction probability of 10 to 95 percent (Figure 4C).

## 4 Discussion

In the present study, we used MRI as the object of feature extraction for predicting benign and malignant testicular lesions. Among all methods, the XGBoost classifier achieved best predictive performance, and the results revealed that machine learning models established based on radiomics features were able to differentiate benign from malignant testicular lesions.

Currently, MRIs serve as powerful tools that offer valuable insights into the characterization of various pathologies. The differentiation of testicular lesions, particularly between benign and malignant lesions, presents significant challenges for clinical experts. For radiologists, the visual differentiation of testicular lesions in MRI often requires a high level of expertise and experience. In terms of visual differential diagnosis, experts typically rely on certain key characteristics observed in MRI. The integration of machine learning models, particularly those employing radiomic analysis, aims to overcome these challenges by quantitatively analyzing a wider range of features than what the human eye can discern. These advanced techniques offer promising avenues for improving diagnostic accuracy; they are intended to complement the expert judgment of clinical professionals. MRI-based radiomics models are emerging as an innovative approach to aid clinical decision-making. Several previous studies illustrate the efficacy and superior performance of these models. For instance, Zhang et al. (22) carried out a comparative analysis between traditional models and MRI-based radiomics models for diagnosing divergent carotid plaques. The outcomes indisputably denoted enhanced diagnostic performance by the radiomics model. Furthermore, the contribution of the AdaBoost classifier was substantial in differentiating low-grade gliomas from glioblastoma peritumoral regions relying on MRI radiomics (23). In this study, we observed a strong association and impressive correlation among the predicted probabilities of the boosting algorithms, such as gradient boosting (GB), AdaBoost, and extreme gradient boosting (XGBoost), across all examined cases. This signifies their potential for effectively distinguishing between benign and malignant lesions based on multidimensional radiomic data. Furthermore, we unveiled a noteworthy finding: the random forest (RF) model and these boosting algorithms yielded correlation coefficients equal to or higher than 0.88. This talent of the integrated algorithm to capture complex relationships between various features is well reflected in its superior performance. Interestingly, the logistic regression (LR) model was found to have a high correlation coefficient with the RF model. This emphasizes that classical models can powerfully differentiate between benign and malignant tumors. Hence, we should not undermine their potential while exploiting the power of advanced algorithms. Overall, the robust performance of the MRI-based radiomics models in our study, alongside findings from prior research, proposes a promising paradigm for future clinical applications. Particularly for the classification and diagnosis of diverse pathologies, these models could influence a shift from conventional diagnostic methods towards a more integrated and personalized approach.

In this study, the superior performance of XGBoost may be attributed to its gradient boosting framework, which inherently minimizes exponentially the discrepancy between predicted and true outcomes at each iteration. It’s this boosting feature that makes it a robust and reliable algorithm for modeling complex patterns and predicting outcomes in healthcare data. Moreover, the significant findings of our study showcase the potential of employing machine learning models built on the basis of radiomic features in clinical radiology. Unlike conventional assessment methods, which rely heavily on subjective impressions or labor-intensive quantitative volumetric analysis, machine learning offers an objective and systematic approach to medical imaging evaluation. By leveraging robust algorithms, it allows for high-throughput detection and quantification of pertinent images’ features, offering reproducible and unbiased results. At its core, our findings highlight a paradigm shift in the evaluation of testicular lesions. The coupling of radiomics, machine learning, and, specifically, the use of the XGBoost algorithm underscores the emergence of a new era in clinical diagnostics. Interestingly, our study not only documents an improved method for predicting benign and malignant lesions but also sets a benchmark for future research to further optimize these prediction models, thereby enhancing our understanding and management of testicular diseases.

Correct noninvasive preoperative diagnosis is critically important for proper clinical decision-making and devising appropriate surgical plans, as it seeks to prevent unnecessary orchiectomy and enhance the quality of patient care. MRI has emerged as a promising imaging modality, exhibiting valuable radiomic features particularly relevant to testicular germ cell tumors (24, 25). As the body of literature in this area advances, a greater understanding of these radiomic characteristics can refine diagnostic accuracy and impact clinical practice. Zhang et al. (26) demonstrate the potential of T2-weighted imaging (T2WI)-based radiomics for differentiating seminomas from non-seminomas, yielding an impressive area under the curve (AUC) score of 0.979. In comparison to Zhang’s study, our investigation benefits from a larger sample size and demonstrates substantial diagnostic performance by leveraging sophisticated machine learning algorithms. This improved methodology adds validity to our results and bolsters the case for the incorporation of MRI-based machine learning models in disease diagnosis. Similarly, He et al. (27) explore the application of MRI-based radiomic models for distinguishing benign and malignant prostate lesions. The study reports AUCs of 0.775 (T2WI) and 0.863 (apparent diffusion coefficient, ADC) for models based on single sequences. More notably, the integration of clinical characteristics enhances lesion discrimination capabilities, indicating the potential for combining radiomic data with patient profiling to further optimize diagnostic performance. The convergence of MRI and machine learning in these studies represents a paradigm shift in diagnostic approaches, signifying the growing importance of noninvasive and accurate methods in clinical practice. By transcending traditional, subjective assessments, machine-learning-assisted MRI has the potential to provide robust, reproducible, and data-driven insights with the added advantage of efficient, high-throughput analysis.

Notably, other imaging domains, such as ultrasound, may also contribute to distinguishing benign and malignant testicular tumors. Ultrasound imaging is a first-line, non-invasive diagnostic tool used in the evaluation of testicular tumors. It allows us to observe variations in size, shape, and location and to detect any discrete lesions, which can help guide clinical management. Typically, benign testicular tumors are well-defined, have homogeneous consistency, and may exhibit a halo of hypervascularity if there is inflammation or cystic changes. Various benign tumors, such as Leydig cell tumors, Sertoli cell tumors, and granulosa cell tumors, can be identified based on these characteristics. Conversely, malignant testicular tumors often present with a heterogeneous echo texture due to areas of necrosis, hemorrhage, or calcification. Growth patterns, vascularization, and the presence of metastatic tumors in the abdomen or pelvis seen on ultrasound can help identify malignant conditions such as seminomas and non-seminomatous germ cell tumors. Isidori et al. (28) investigated the accuracy of non-enhanced ultrasound combined with enhanced ultrasound in distinguishing benign and malignant lesions of ≤1.5 cm in the testes. Their results demonstrated that the combination of unenhanced and contrast-enhanced US achieved high accuracy in the diagnosis of small testicular malignancies (area under the ROC curve performance: 0.927; 95% confidence interval: 0.872, 0.981). This study suggests that the combination of enhanced and non-enhanced ultrasound effectively distinguishes benign and malignant testicular lesions of ≤1.5 cm, compensating for the inferior differentiating ability of non-enhanced ultrasound. However, it should be noted that ultrasound findings alone may not definitively distinguish benign from malignant tumors. Correlation with patient history, physical examination, and tumoral markers can further substantiate the diagnosis.

The current study emphasizes the paramount need for an accurate prognosis of testicular lesions in the pursuit of limiting false-negative results, as wrongful identification can pose a significant risk for patients. Orchiectomy stands as the conventional method of treatment for presumptive malignant testicular masses; however, the potential for error underscores the importance of discerning between benign and malignant testicular lesions. Misdiagnosis can result in unnecessary surgical intervention or postpone necessary treatment, thereby influencing patient outcomes and quality of life. Each patient presents a unique probability of predicting malignant testicular lesions, thereby underscoring individual-based therapeutic planning. In our quest to strike a risk-benefit balance, decision curve analysis (DCA) holds immense promise as a means to offer quantitative reference values that can inform the treatment strategy. This study incorporated DCA as a key component in our evaluation methodology for the listed model’s prediction results. By presenting a graphical representation of the model’s applicability at varying threshold probabilities, DCA aids in the comprehension of potential benefits against potential harms in decision-making processes. Moreover, it augments the traditional measures of test performance by integrating patient preferences into the analysis. Our model’s performance demonstrated significant consistency with the actual rate of testicular cancer across all cases, as revealed by the calibration plot. In essence, the calibration plot offers a visual demonstration of the model’s predictive qualities in comparison to the ideal prediction. A curve that aligns closely with the 45-degree line infers perfect calibration, whereas deviation from the line implicates over- or underestimation. Thus, the proximity of the presented calibration curve to the real cancer rate supports the robustness of our model in predicting malignant testicular lesions. Moreover, the results of DCA computations signal that our model is generally applicable for a broad scope of threshold probabilities. It accentuates the rigor of the model predictions and manifests its potential adaptability across a spectrum of clinical settings.

This study focused on T2WI for the diagnosis of testicular diseases, as it is a routine and pivotal sequence in testicular MRI protocols. T2WI offers exceptional tissue contrast resolution, which is crucial for accurately delineating testicular lesions and differentiating between various disease types. This technique highlights differences in tissue composition and internal lesion structure, aiding in the identification of features like cystic components and solid areas. While diffusion-weighted imaging (DWI) and dynamic contrast-enhanced (DCE) sequences have diagnostic value, their limited use in clinical practice restricted their inclusion in our analysis.

Our study had several limitations. First and foremost, this study’s reliance on data from a single center limits the scope of its findings. Given the wide spectrum of global health contexts and population dynamics, it should be noted that results derived from a single-center study may not be universally applicable. As a result, our findings should be interpreted with a certain level of caution when extended to other settings with differing population and health system characteristics. Future research could benefit from a multi-center trial, which would allow for a more diverse sampling of patient populations and healthcare settings. This would enhance the generalizability of our findings and further validate the insights we have gleaned from this investigation. Secondly, we must acknowledge the relatively small sample size of our study due to the low incidence of testicular cancer. While this small sample size enabled us to investigate this essential topic, it could nonetheless have affected the statistical power and practicability of our study. Considering this, we propose that future research on this topic strive for larger sample sizes to ensure a more robust analysis of data, gain a nuanced understanding of this cancer variety, and facilitate a more reliable estimate of the examination process’s practicability. Lastly, we have recognized that the usage of the mRMR (minimum redundancy maximum relevance) algorithm could potentially underestimate the importance of features that individually bear limited impact on the targeted outcome but collectively can be highly effective. While the mRMR algorithm serves as a valuable tool for selecting relevant features in a dataset, it may not recognize the cumulative effect of feeble features. Future investigations should consider evaluating alternative methods alongside, or instead of, the mRMR algorithm. Employing different feature selection techniques could potentially give a more holistic view of factors affecting clinical outcomes, thereby enhancing the robustness and reliability of the results.

In summary, despite these limitations, our study provides essential insights into the fight against testicular cancer. Patient prognosis and treatment could be improved through further multi-center studies with larger sample sizes and different statistical methods. Nevertheless, it is vital that future research builds on this foundation and continues to explore these avenues to further advance our understanding and capabilities in combating this disease.

## 5 Conclusion

In conclusion, machine learning models based on MRI could accurately predict benign and malignant testicular lesions in the present study. Compared with a simple machine learning model, the ensemble model may achieve better performance, particularly when using the boosting algorithm represented by XGBoost. Information from a single sequence is limited, prompting the potential combination of different types of images or multiple sequences of a particular kind for machine learning training and prediction in the future. Additionally, integrating different machine learning could enhance predictive effectiveness.

## Data availability statement

The raw data supporting the conclusions of this article will be made available by the authors, without undue reservation.

## Ethics statement

The studies involving humans were approved by Tongji Hospital, Tongji Medical College, Huazhong University of Science & Technology. The studies were conducted in accordance with the local legislation and institutional requirements. The ethics committee/institutional review board waived the requirement of written informed consent for participation from the participants or the participants’ legal guardians/next of kin because written informed consent was waived in view of the retrospective nature of the study and all the procedures being performed were part of the routine examination.

## Author contributions

YF: Formal analysis, Methodology, Writing – original draft, Investigation, Software. ZF: Data curation, Funding acquisition, Investigation, Writing – original draft. LW: Writing – review & editing. WL: Formal analysis, Methodology, Writing – review & editing, Conceptualization. ZL: Writing – review & editing. XM: Writing – review & editing, Data curation, Writing – original draft. JL: Funding acquisition, Resources, Supervision, Writing – review & editing, Project administration. JZ: Funding acquisition, Project administration, Resources, Supervision, Writing – review & editing.
