# Supplementary material for: Comparison and analysis of multiple machine learning models for discriminating benign and malignant testicular lesions based on magnetic resonance imaging radiomics
Source: Front Med (Lausanne). 2023 Dec 21;10:1279622. doi: 10.3389/fmed.2023.1279622 (PMC10768048; doi:10.3389/fmed.2023.1279622)
Supplement: Supplementary file 1 [file Table_1.DOCX]

Supplementary Material

# Supplementary Figures and Tables

**Table S1. Parameters of the MRI protocol.**

| Sequence | T2WI | T1WI | DWI | DCE-MRI |
| --- | --- | --- | --- | --- |
| Technique | Transverse, sagittal, and coronal fast spin-echo | Transverse turbo spin-echo | Transverse single-shot spin-echo | Axial three-dimensional T1-weighted spoiled gradient echo. |
| TR/TE (ms) | 6500 - 6870 /104 | 750/13 | 5300/78 | 5.08/1.77 |
| Thickness (mm) | 3 - 5 | 3 - 5 | 3 - 5 | 3.5 |
| Intersection gap (mm) | 0 - 0.5 | 0 - 0.5 | 0 - 0.5 | 0.7 |
| FOV (mm) | 180×180 | 300×300 | 220×176 | 260×260 |
| Matrix | 384 × 320 | 320×240 | 90×90 | 192×154 |
| Parallel Imaging Factor | 2 | 2 | NA | 2 |
| Flip Angle (degree) | 160 | 160 | 90 | 15 |
| b-values (s/mm^2^) | NA | NA | 50, 1000 | NA |

T2WI: T2-weighted imaging; T1WI: T1-weighted imaging; DCE-MRI: dynamic contrast-enhanced magnetic resonance imaging; DWI: diffusion-weighted imaging; TR: repetition time; TE: echo time; FOV: field of view; NA: no application.

**Table S2 Filters selected for the present study.**

| **Filter Class** | **Filter** | **count** |
| --- | --- | --- |
| Laplacian of Gaussian | LoG-sigma 2.0  LoG-sigma 3.0 | 2 |
| Wavelet | Wavelet-LLL  Wavelet-HLL  Wavelet-LHL  Wavelet-HLH  Wavelet-HHL  Wavelet-LLH  Wavelet-LHH  Wavelet-HHH | 8 |
| Square | Square | 1 |
| Square Root | Square Root | 1 |
| Logarithm | Logarithm | 1 |
| Exponential | Exponential | 1 |
| Gradient | Gradient | 1 |
| Local Binary Pattern (LBP) 3D | LBP-3D-m1  LBP-3D-m2  LBP-3D-k | 3 |

**Table S3 Feature classes extracted from images.**

| **Feature Class** | **Feature Name** | **Count** |
| --- | --- | --- |
| First Order Features | 10Percentile  90Percentile  Energy  Entropy  InterquartileRange  Kurtosis  Maximum  MeanAbsoluteDeviation  Mean  Median  Minimum  Range  RobustMeanAbsoluteDeviation  RootMeanSquared  Skewness  TotalEnergy  Uniformity  Variance | 18 |
| Shape Features (3D) | Flatness  LeastAxisLength  MajorAxisLength  Maximum2DDiameterColumn  Maximum2DDiameterRow  Maximum2DDiameterSlice  Maximum3DDiameter  MeshVolume  MinorAxisLength  Sphericity  SurfaceArea  SurfaceVolumeRatio  VoxelVolume | 14 |
| Gray Level Co-occurrence Matrix (GLCM) | Autocorrelation  ClusterProminence  ClusterShade  ClusterTendency  Contrast  Correlation  DifferenceAverage  DifferenceEntropy  DifferenceVariance  Id  Idm  Idmn  Idn  Imc1  Imc2  InverseVariance  JointAverage  JointEnergy  JointEntropy  MCC  MaximumProbability  SumAverage  SumEntropy  SumSquares | 24 |
| Gray Level Run Length Matrix (GLRLM) | GrayLevelNonUniformity  GrayLevelNonUniformityNormalized  GrayLevelVariance  HighGrayLevelRunEmphasis  LongRunEmphasis  LongRunHighGrayLevelEmphasis  LongRunLowGrayLevelEmphasis  LowGrayLevelRunEmphasis  RunEntropy  RunLengthNonUniformity  RunLengthNonUniformityNormalized  RunPercentage  RunVariance  ShortRunEmphasis  ShortRunHighGrayLevelEmphasis  ShortRunLowGrayLevelEmphasis | 16 |
| Gray Level Size Zone Matrix (GLSZM) | GrayLevelNonUniformity  GrayLevelNonUniformityNormalized  GrayLevelVariance  HighGrayLevelZoneEmphasis  LargeAreaEmphasis  LargeAreaHighGrayLevelEmphasis  LargeAreaLowGrayLevelEmphasis  LowGrayLevelZoneEmphasis  SizeZoneNonUniformity  SizeZoneNonUniformityNormalized  SmallAreaEmphasis  SmallAreaHighGrayLevelEmphasis  SmallAreaLowGrayLevelEmphasis  ZoneEntropy  ZonePercentage  ZoneVariance | 16 |
| Gray Level Dependece Matrix (GLDM) | DependenceEntropy  DependenceNonUniformity  DependenceNonUniformityNormalized  DependenceVariance  GrayLevelNonUniformity  GrayLevelVariance  HighGrayLevelEmphasis  LargeDependenceEmphasis  LargeDependenceHighGrayLevelEmphasis  LargeDependenceLowGrayLevelEmphasis  LowGrayLevelEmphasis  SmallDependenceEmphasis  SmallDependenceHighGrayLevelEmphasis  SmallDependenceLowGrayLevelEmphasis | 14 |
| Neighboring Gray Tone Difference Matrix (NGTDM) | Busyness  Coarseness  Complexity  Contrast  Strength | 5 |

**Table S4 Average values of performance metrics determined using the training cohort 10 times.**

| Model | AUC | Sensitivity | Specificity | Accuracy | Average Precision | NPV | PPV |
| --- | --- | --- | --- | --- | --- | --- | --- |
| QDA | 0.905 | 0.654 | 1.000 | 0.775 | 0.952 | 0.609 | 1.000 |
| LR | 0.826 | 0.654 | 0.893 | 0.738 | 0.908 | 0.581 | 0.919 |
| DT | 0.796 | 0.923 | 0.643 | 0.825 | 0.822 | 0.818 | 0.828 |
| SVM | 0.897 | 0.904 | 0.786 | 0.863 | 0.937 | 0.815 | 0.887 |
| KNN | 0.852 | 0.538 | 1.000 | 0.700 | 0.894 | 0.538 | 1.000 |
| GaussianNB | 0.828 | 0.731 | 0.821 | 0.763 | 0.903 | 0.622 | 0.884 |
| RF | 0.908 | 0.856 | 0.832 | 0.848 | 0.948 | 0.759 | 0.905 |
| GB | 0.936 | 0.788 | 0.964 | 0.850 | 0.964 | 0.711 | 0.976 |
| AdaBoost | 0.923 | 0.712 | 1.000 | 0.813 | 0.954 | 0.651 | 1.000 |
| XGBoost | 0.987 | 0.942 | 0.964 | 0.950 | 0.994 | 0.900 | 0.980 |
